# A Temporary Immersion System to Improve *Cannabis sativa* Micropropagation

**DOI:** 10.3389/fpls.2022.895971

**Published:** 2022-06-23

**Authors:** Saleta Rico, José Garrido, Conchi Sánchez, Carlos Ferreiro-Vera, Verónica Codesido, Nieves Vidal

**Affiliations:** ^1^Misión Biológica de Galicia- Sede Santiago de Compostela, MBG-CSIC, Departamento Producción Vegetal, Santiago de Compostela, Spain; ^2^Phytoplant Research S.L.U, Departamento Hibridación y Cultivo, Rabanales 21-Parque Científico Tecnológico de Córdoba, Calle Astrónoma Cecilia Payne, Córdoba, Spain

**Keywords:** bioreactors, liquid medium, mass propagation, Plantform™, RITA, sucrose

## Abstract

The aim of this study was to propagate axillary shoots of *Cannabis sativa* L. using liquid medium in temporary immersion bioreactors. The effect of immersion frequency (3 or 6 immersions per day), explant type (apical or basal sections), explant number (8, 10, and 16 explants), mineral medium (Murashige and Skoog half-strength nitrates, *β*-A and *β*-H, all supplemented with 2-μM metatopoline), sucrose supplementation (2, 0.5, and 0% sucrose), culture duration (4 and 6 weeks), and bioreactor type (RITA® and Plantform™) were investigated. As a result, we propose a protocol for the proliferation of cannabis apical segments in RITA® or Plantform™ bioreactors. The explants (8 per RITA® and 24 per Plantform™) are immersed for 1 min, 3 times per day in *β*-A medium supplemented with 2-μM metatopoline and 0.5% of sucrose and subcultured every 4 weeks. This is the first study using temporary immersion systems in *C. sativa* production, and our results provide new opportunities for the mass propagation of this species.

## Introduction

*Cannabis sativa L*. (Cannabaceae) is a high-demand multipurpose crop with medicinal, nutritional, industrial, recreational, and agricultural uses (Hesami et al., 2020; Kovalchuk et al., 2020). Cannabis is an annual herb, predominantly dioecious and occasionally monoecious (Chandra et al., 2017). Cannabis plants can be divided into the following two main groups according to its percent of psychoactive cannabinoids based on dry weight: Plants with flowers with <0.2–0.3% Δ9-tetrahydrocannabinol (the exact threshold depends on the country) are classified as hemp, and plants that produce 0.2–0.3% or higher are categorized as marijuana (Oultram et al., 2021).

Cannabis leaves and flowers produce a broad spectrum of biologically active secondary metabolites, seeds are a source of nutritious oil and protein, and the stem contains two types of fiber serving as feedstock for a variety of bio-based consumer goods (Adhikary et al., 2021). During the last decade, the industrial applications of cannabis in textiles, paper, building materials, cosmetics, and foods, as well as pharmacological properties have been widely studied and supported (Hesami et al., 2021a). Cannabinoids and cannabinoid-containing products have created a new market that is growing exponentially (Valleriani, 2020). The recent cannabis legalization in many regions for medicinal and recreational purposes and the establishment of a legal market for cannabinoids has promoted systematic research and use of this plant (Aliferis and Bernard-Perron, 2020; Adhikary et al., 2021). As the demand for these products grow, there is an increasing interest in developing biotechnologies and improved cultivation techniques for effective propagation (Monthony et al., 2021b).

Dioecy and regulation of cannabis plants make conventional breeding methods difficult, time consuming, costly, and laborious (Hesami et al., 2021a). Traditionally, cannabis has been propagated from seed and stem cuttings. Vegetative propagation maintains genetic integrity and uniformity among the plants, which is not possible with seed propagation. In general, cannabis is relatively easy to root, and large numbers of plants can be produced from a single mother plant (Campbell et al., 2019) but significant amounts of space are needed. This approach requires the maintenance of mother plants in a vegetative state and free of pests and diseases (Adhikary et al., 2021; Monthony et al., 2021b).

Micropropagation methods providing uniform explants and better control of environmental conditions may represent an alternative to conventional (*in vivo*) clonal propagation and germplasm maintenance of *C. sativa. In vitro* culture allows the rapid production of a great number of genetically identical plants in a very small space and with efficient use of resources including time. These techniques may help for long-term genetic preservation as well as they can produce and maintain disease-free plants. However, the current micropropagation protocols for the most *in vitro* plants including cannabis are labor-intensive and costly, and an automation is difficult. These techniques require a large number of containers, semi-solid medium with the manual handling of the tissues in aseptic conditions. The widespread commercial application of micropropagation will only be economically possible when technologies that automate these processes are developed.

Liquid medium in micropropagation is considered the ideal solution for large scale propagation to reduce production costs and to introduce automation (Aitken-Christie, 1991). The cultivation systems that use liquid medium provide more uniform conditions and several advantages over gelled medium in micropropagation systems as follows: It lowers the plantlet production costs due to reduced agar use; the media can be easily renewed without changing the container; sterilization by microfiltration is possible; and cleaning the containers after the culture period is much easier. Compared to cultivation in semi-solid media, much larger containers can be used, and the transfer times can be shortened. However, liquid culture has some technical problems such as asphyxia and hyperhydricity (Etienne and Berthouly, 2002) as well as potential contamination issues.

Temporary immersion systems (TIS) were developed to resolve these problems (Steingroewer et al., 2013). The atmosphere in TIS can be renewed; thus, reducing disorders such as asphyxia and hyperhydricity. The frequency and duration of immersion, liquid medium volume, number of explants, aeration, and forced ventilation are critical factors to optimize the micropropagation technique using TIS (Etienne and Berthouly, 2002). The regulation of time between the immersions and the exposure periods can help in reducing the problem of hyperhydricity (Albarrán et al., 2005). The most popular bioreactors for TIS include the following: Twin–Flask system, Ebb-and-Flow, RITA®, Thermo-photo-bioreactor, TIS, and Plantform™ (Georgiev et al., 2014). Recently, several studies have shown that TIS have numerous advantages as regards the semi-solid methods (Vidal and Sánchez, 2019). These systems have been successfully used for micropropagation of *Stevia rebaudiana* (Melviana et al., 2021), *Colocasia esculenta* L. Schott (Mancilla-Álvarez et al., 2021), *Agave angustifolia* (Monja-Mio et al., 2021), *Rosmarinus officinalis* L. (Villegas-Sánchez et al., 2021), *Dracocephalum forrestii* (Weremczuk-Jezyna et al., 2020), *Guarianthe skinneri* (Leyva-Ovalle et al., 2020), and the number is increasing steadily. In our laboratory, we have developed protocols using bioreactor propagation systems with liquid medium for axillary shoots of chestnut, alder and willow (Vidal et al., 2015; Cuenca et al., 2017; Regueira et al., 2018; San José et al., 2020; Gago et al., 2021), and somatic embryos of *Quercus robur* (Mallón et al., 2012, 2013).

Some micropropagation protocols have been developed for *C. sativa* (Adhikary et al., 2021; Hesami et al., 2021a; Monthony et al., 2021b). However, to the best of our knowledge, there is only a congress communication referring the study of temporary immersion bioreactor systems (Lata et al., 2010). Bioreactors can help overcome proliferation difficulties with some cannabis genotypes, including rooting and acclimation issues, as reported for recalcitrant species (Vidal and Sánchez, 2019). In addition, they can reduce the cost of large-scale propagation and facilitate photoautotrophy (Xiao et al., 2011; Adhikary et al., 2021).

The aim of this study was to develop an efficient protocol for culturing cannabis shoots of different genotypes by temporary immersion in liquid medium. The effect of immersion frequency, explant type, number of explants, medium, sucrose supplementation, duration of subculture, and bioreactor type were evaluated in relation to shoot quality and proliferation rates.

## Materials and Methods

### Plant Material and Culture Conditions

Three cannabis genotypes registered in the European Community Plant Variety Office (CPVO; https://cpvo.europa.eu/en) with different cannabinoid content were used: Beatriz (App. No. 20170146), Mati (App. No. 20170147), and Moniek (App. No. 20160114). These varieties were provided by Phytoplant Research SLU (Spain), a company specializing in the development of commercial scale production of medicinal plants. These genotypes were established *in vitro* from axillary buds harvested from young shoots and maintained in 500 ml glass jars (6 explants per jar) with 70 ml of *β*-A medium (Codesido et al., 2020). This media was supplemented with 2-μM metatopoline (MT), as reported by Lata et al. (2016), for a wide range of cannabis genotypes, with 2% sucrose (w/v) and 0.8% Bacto™ agar (w/v) as gelling agent. The media pH was adjusted to 5.7 before autoclaving at 120°C for 20 min. All chemicals used in this study were purchased from Duchefa Biochemie (The Netherlands) except the *β*-media, which was provided by Phytoplant Research SLU (Spain), and Bacto™ agar, purchased from Difco (Becton Dickinson & Co.). The cultures were incubated in a growth chamber with a 16-h photoperiod provided by coolwhite fluorescent lamps (50–60 μmol m^−2^s^−1^) at 25°C light/20°C dark (standard conditions) and subcultured every 6 weeks.

### Micropropagation in Liquid Medium

**Figure 1:**
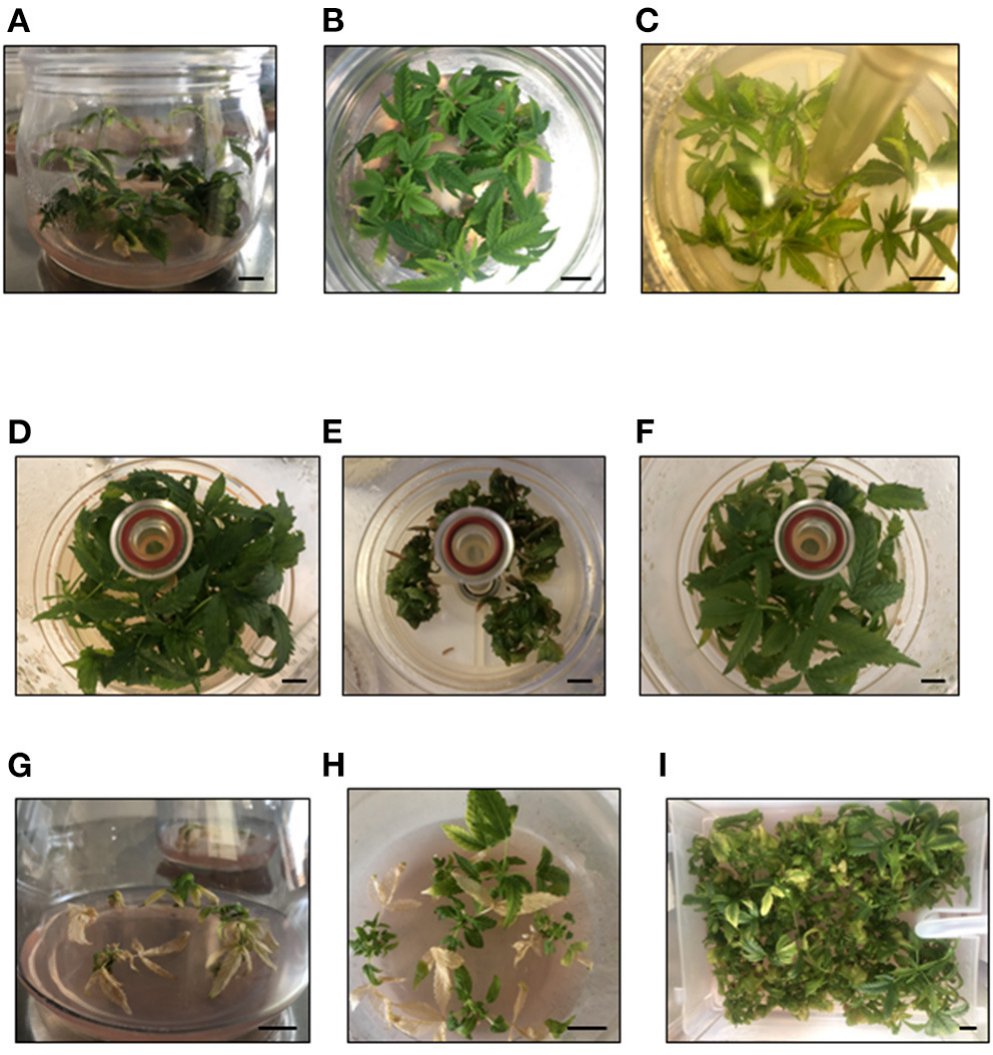
Mati **(A)** and Beatriz **(B)** genotypes cultured in *β*-A semi-solid medium with 2% sucrose. Beatriz genotype cultured in RITA® with MS ½ N with 2% sucrose **(C)**, *β-*A with 2% sucrose **(D)**, MS–½N with 0.5% sucrose **(E)**, and *β*-A with 0.5% sucrose **(F)**. Mati **(G)** and Beatriz **(H)** genotypes cultured in *β*-A semi-solid medium with 0.5% sucrose, Beatriz genotype **(I)** cultured in Plantform™ with *β*-A medium and 0.5% sucrose. Bars, 10 mm.

The initial cannabis explants used for TIS were 15-mm apical sections derived from the shoots growing in semi-solid medium (Figures 1A,B). The experiments were carried out in the following two types of commercial bioreactors: RITA® bioreactors (www.vitropic.fr) were used to study the main factors affecting cannabis proliferation in liquid medium and the best conditions derived from these experiments were applied to Plantform^TM^ bioreactors (www.plantform.se). Both types of bioreactors were used following the instructions of the manufacturers. Each RITA® contained 150 ml of liquid medium and each Plantform^TM^ contained 600 ml of liquid medium. The following three media were used: One based on the Murashige and Skoog (MS) formulation (Murashige and Skoog, 1962), namely, MS medium with half-strength nitrates (MS–½N) including vitamins, and two media based on Formula *β* (Casano and Grassi, 2009). These later media, designated as *β*-H and *β*-A did not include vitamins (Codesido et al., 2020). All media contained 2-μM MT and 2% sucrose (except for the experiment of sucrose supplementation). The liquid media was autoclaved, then added to the containers. The bioreactors and the 0.22-μm hydrophobic filters were autoclaved separately. Inoculated bioreactors were incubated for 4 weeks under the standard conditions previously described for semi-solid cultures.

The following parameters were evaluated in the application of TIS to cannabis cultures: (i) frequency of immersion (3 or 6 immersions per day, duration 1 min), (ii) number of explants per RITA® bioreactor (8, 10, or 16), (iii) culture medium (MS–½N, *β*-H, and *β*-A), (iv) duration of subculture (4 or 6 weeks), (v) sucrose supplementation (0, 0.5, and 2%), (vi) type of explant (apical or basal sections), and (vii) type of bioreactor (RITA® and Plantform™).

The following variables were assessed: Total number of shoots longer than 15 mm produced by each explant (NS); multiplication coefficient (MC), which was defined as the number of new segments of 15 mm valid for subculturing from each initial explant; shoot length (SL), which was the length of the longest shoot per explant; and hyperhydricity (H), which was calculated as the percentage of hyperhydric shoots.

### Experimental Design and Statistical Analysis

The data correspond to four replicates per treatment and eight explants per replicate, except in the experiment evaluating type of bioreactor where three replicates per treatment and 24 explants per replicate were used. The data were submitted to Levene's test to verify the homogeneity of variances, then subjected to Student's *t*-test or analysis of variance (ANOVA) followed by comparison of group means (Tukey-*b* test). When an interaction between two factors was indicated by the two-way ANOVA, Bonferroni's adjustment was applied to detect simple main effects in multiple *post hoc* comparisons. A *p* ≤ 0.05 was considered statistically significant. The percentage data were subjected to arcsine transformation prior to analysis and non-transformed data are presented in the results. Statistical analyses were performed using SPSS 23.0 (IBM).

## Results

### Effect of Immersion Frequency in RITA® Vessels

**Figure 2:**
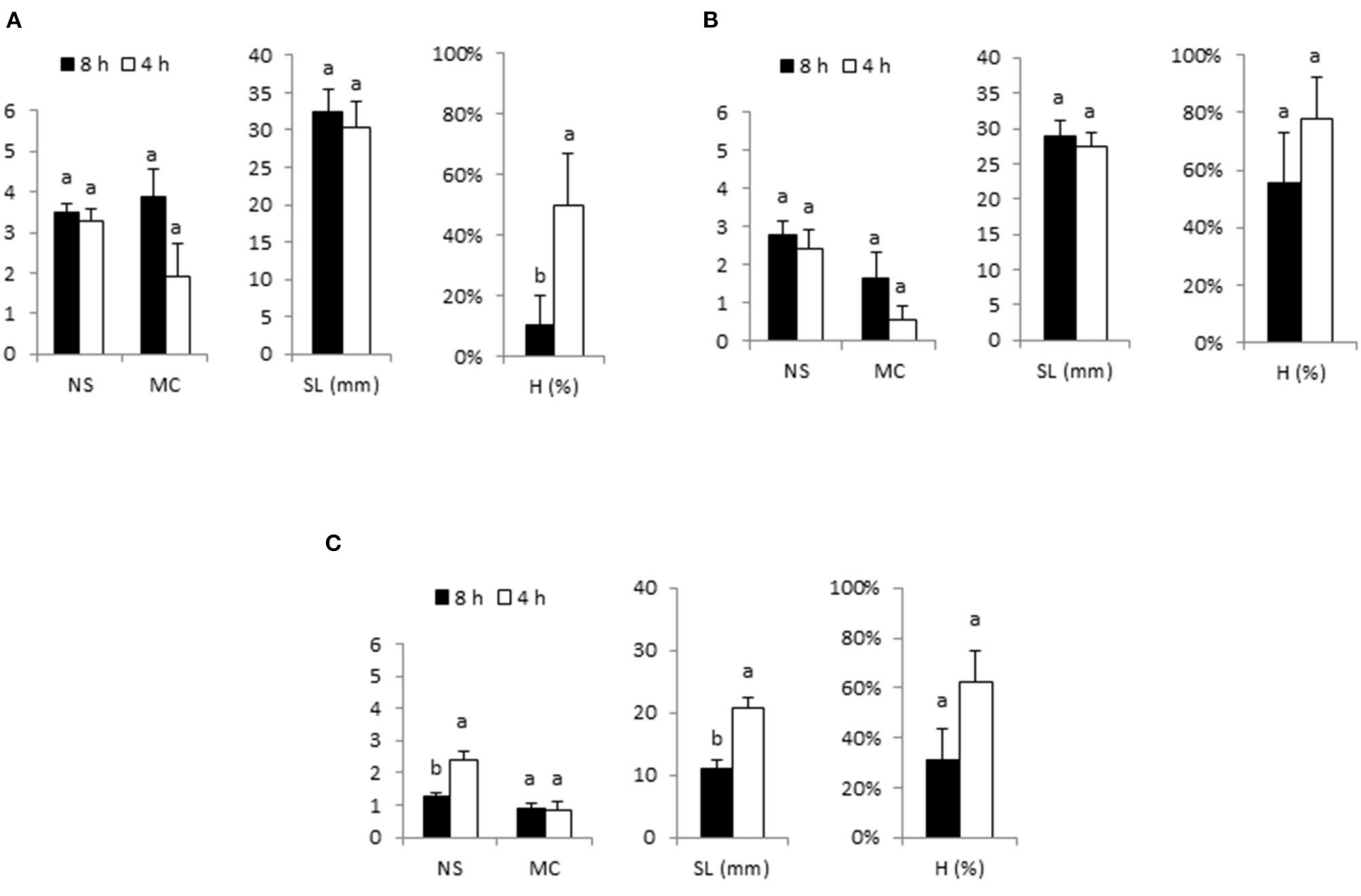
Effect of immersion frequency (every 8 or 4 h) on proliferation rates of apical sections of Beatriz **(A)**, Moniek **(B)**, and Mati **(C)** genotypes cultured in RITA® vessels. Explants were grown in MS medium with half-strength nitrates and 2-μM metatopoline. Immersion duration was set at 1 min. Ten explants/RITA® were used for Beatriz and Moniek and 16 explants/RITA® for the Mati genotype. The data were recorded after 4 weeks of culture. The values represent the mean ± standard error. For each variable, different letters indicate significant differences at *p* < 0.05. NS, number of shoots per explant; MC, multiplication coefficient; SL, length of the longest shoot (mm); H, percentage of hyperhydric shoots.

The results of immersing apical sections of cannabis for 1 min every 8 or 4 h in RITA® bioreactors (3 or 6 times per day) are shown in Figure 2. For genotypes Beatriz and Moniek, increasing the frequency of immersion from once every 8 h to once every 4 h led to similar NS and SL. However, as H was increased, not all new shoots could be used for multiplication, and a decrease in MC was observed (Figures 2A,B). In the case of Mati genotype, increasing the immersion frequency led to a significant increase in NS and SL, but as happened with Beatriz and Moniek, the higher occurrence of H (63%) limited the utility of this treatment (Figure 2C). The shoots were therefore immersed for 1 min every 8 h (3 times per day) in subsequent experiments.

### Effect of the Number of Explants

**Figure 3:**
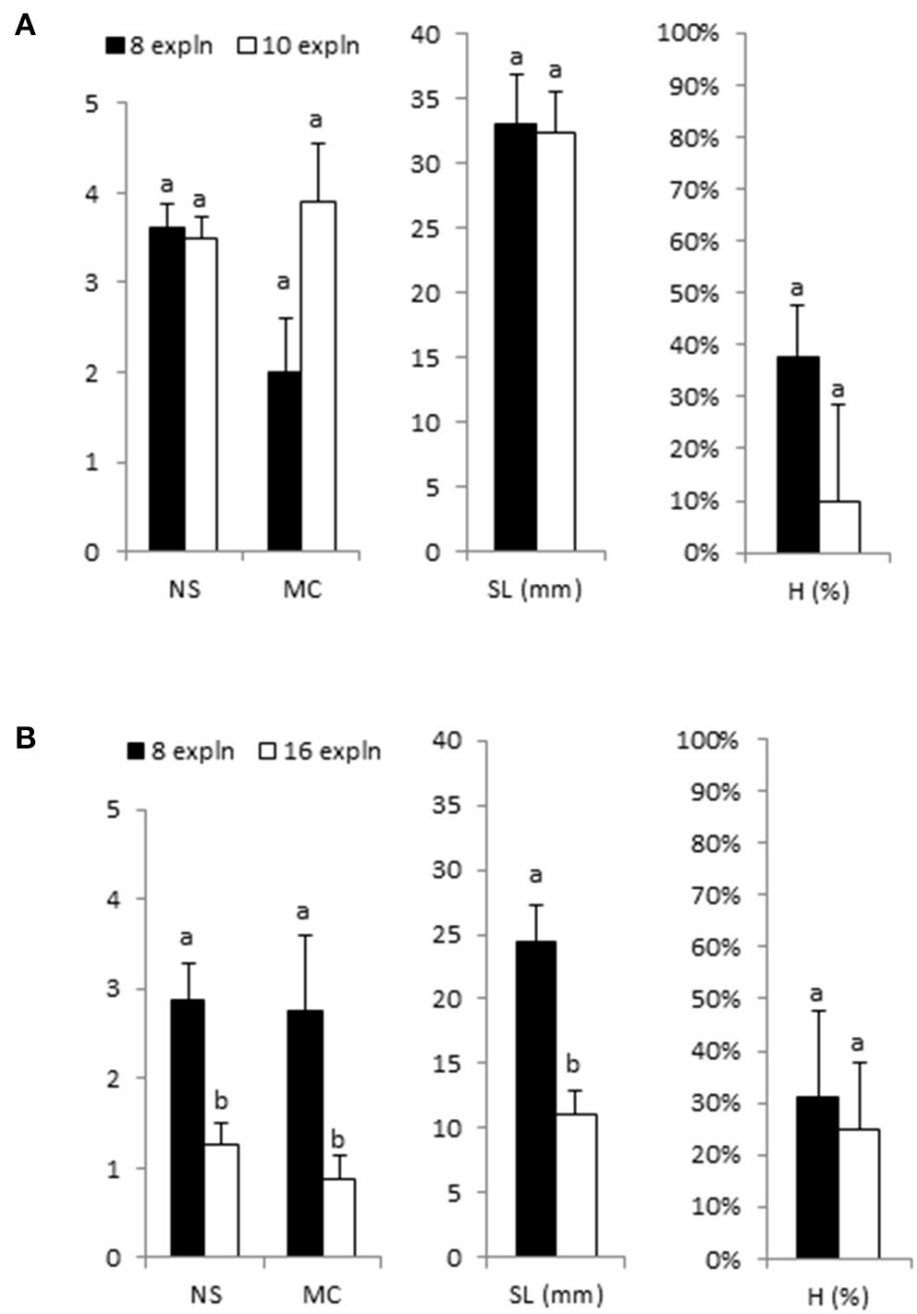
Effect of initial explant number (8, 10, and 16 apical sections) on growth parameters of shoots of Beatriz **(A)** and Mati **(B)** genotypes cultured in RITA® vessels in MS medium with half-strength nitrates and 2-μM metatopoline. The explants were immersed for 1 min every 8 h. The data were recorded after 4 weeks of culture. The values represent the mean ± standard error. For each variable, different letters indicate significant differences at *p* < 0.05. NS, number of shoots per explant; MC, multiplication coefficient; SL, length of the longest shoot (mm); H, percentage of hyperhydric shoots.

Explant densities of 8–10 apical sections/bioreactor were suitable for the proliferation of cannabis. For Beatriz, similar results were obtained with 8 and 10 explants/bioreactor (Figure 3A). However, to double the number of explants for the Mati genotype negatively affected proliferation and shoot quality, more and longer shoots were produced by using 8 instead of 16 initial explants (Figure 3B). Therefore, 8 explants per RITA® were used in further experiments for all genotypes.

### Effect of Culture Medium, Duration of Subculture, and Sucrose Supplementation

The culture medium effects were investigated in the following three experiments studying: (i) the effect of culturing 3 cannabis genotypes for 4 weeks in different media supplemented with 2% sucrose, (ii) interaction between the culture medium and duration of subculture, and (iii) interaction between the culture medium and sucrose supplementation. In all these experiments, eight apical sections/bioreactor were used as initial explants.

The results of culturing Beatriz, Mati, and Moniek genotypes in RITA® for 4 weeks with 2% sucrose are shown in Table 1. For the three genotypes, Formula *β*-A yielded longer shoots and less hyperhydricity than the MS formulation and consequently higher multiplication coefficients were obtained (Table 1 and Figures 1C,D). Using Moniek, in which two media based on Formula *β* were compared, *β*-A resulted in a slightly better performance than *β*-H medium, although the differences were not significant (Table 1).

**Table 1:** Effect of the culture medium (MS–½N, *β*-A, and *β*-H) on the proliferation of apical sections of Beatriz, Mati, and Moniek shoots cultured in RITA® vessels.

***Genotype***	**Medium**	**NS**	**SL (mm)**	**MC**	**%H**
*Beatriz*	MS–½N	3.50 ± 0.22 a	32.40 ± 3.10 b	3.90 ± 0.65 b	10 ± 10 a
	*β*-A	3.75 ± 0.25 a	50.37 ± 0.49 a	7.37 ± 0.56 a	0 ± 0 a
*Mati*	MS–½N	2.12 ± 0.18 b	22.62 ± 1.27 b	0.00 ± 0.00 b	100 ± 0 a
	*β*-A	4.00 ± 0.26 a	36.37 ± 1.95 a	2.69 ± 0.74 a	43 ± 12 b
*Moniek*	MS–½N	2.61 ± 0.29 b	28.20 ± 1.44 b	1.11 ± 0.39 b	66 ± 11 a
	*β*-A	4.00 ± 0.29 a	48.50 ± 4.59 a	4.78 ± 1.01 a	21 ± 11 b
	*β*-H	3.43 ± 0.65 ab	39.28 ± 5.47 a	3.43 ± 1.30 ab	28 ± 18 b

*All media were supplemented with 2-μM metatopoline. Explants (8 per vessel) were immersed for 1 min every 8 h. The data were recorded after 4 weeks of culture. The values represent the mean ± standard error. For each genotype and variable, means indicated by the same letter are not significantly different (p < 0.05). NS, Number of shoots per explant; SL, length of the longest shoot (mm); MC, multiplication coefficient; %H, percentage of hyperhydric shoots*.

**Figure 4:**
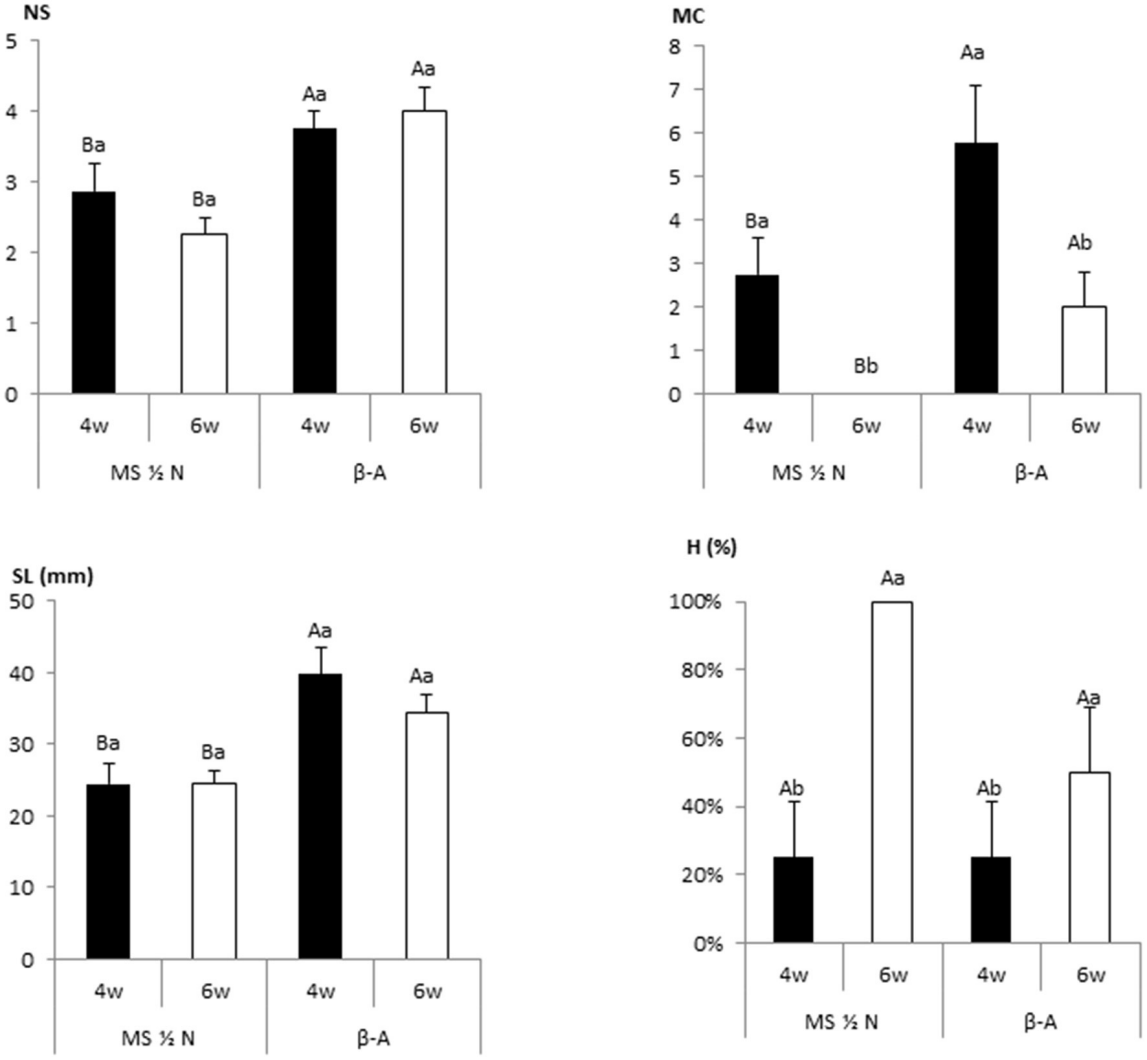
Effect of the culture duration (4 or 6 weeks) on the growth of 8 Mati apical sections cultured in RITA® vessels with MS medium with half-strength nitrates and *β*-A medium. All media were supplemented with 2-μM metatopoline. The data recorded after 4 or 6 weeks of culture. The values represent the mean ± standard error. For each variable, different uppercase letters indicate significant differences in relation to the medium, and different lowercase letters indicate significant differences in relation to the duration of the culture (*p* < 0.05). NS, number of shoots per explant; MC, multiplication coefficient; SL, length of the longest shoot (mm); H, percentage of hyperhydric shoots.

To study the possible interaction between the culture medium and the duration of the subculture Mati shoots were cultured for 4 or 6 weeks in Formula *β*-A and the MS formulation (Figure 4). Media composition significantly affected NS, MC, and SL, and subculturing at 4 weeks produced more proliferation and less hyperhydricity than a 6-week subculture period for the two media tested. No significant interaction between the two variables was detected, with *p*-values of 0.172, 0.328, 0.677, and 0.105 for NS, MC, SL, and H, respectively. The high proliferation rates obtained with liquid medium after 4 weeks of culture, allowed shortening the subculture cycle by 2 weeks relative to the conventional semi-solid medium system, and the 4-week cycle was used thereafter in TIS.

**Figure 5:**
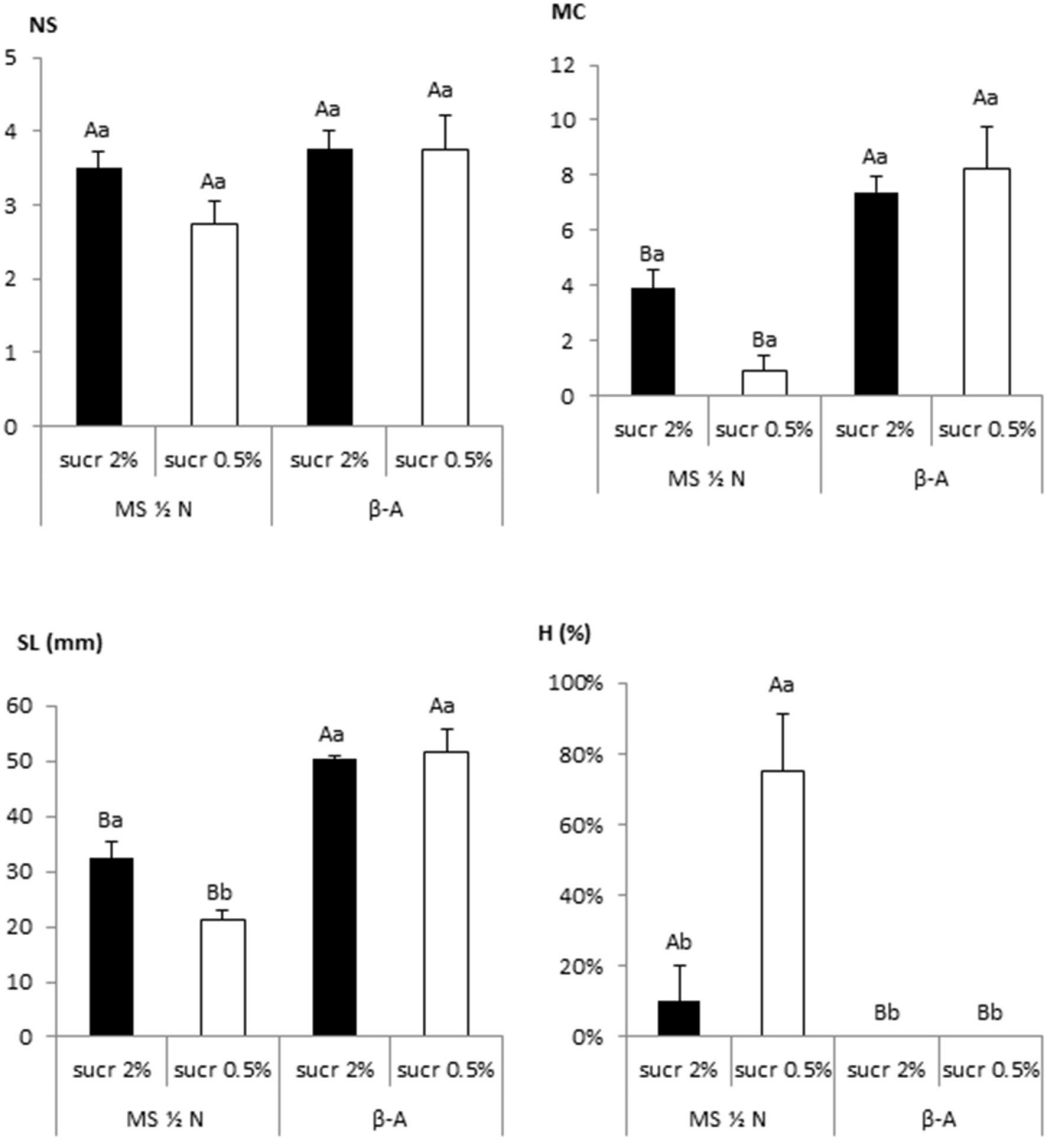
Effect of sucrose and mineral medium on the growth of 8 apical sections of Beatriz genotype cultured in RITA® vessels. Explants were grown in MS medium with half-strength nitrates and in *β*-A medium, both supplemented with 2-μM metatopoline and 2 or 0.5% sucrose. The data were recorded after 4 weeks of culture. The values represent the mean ± standard error. For each variable, different uppercase letters indicate significant differences in relation to the medium, and different lowercase letters indicate significant differences in relation to sucrose supplementation (*p* < 0.05). NS, number of shoots per explant; MC, multiplication coefficient; SL, length of the longest shoot (mm); H, percentage of hyperhydric shoots.

The effect of culturing Beatriz explants for 4 weeks in MS–½N or *β*-A with two sucrose concentrations (2 and 0.5%) is shown in Figure 5. The reduction in sucrose supply adversely affected shoot growth cultured with MS–½N, as reflected by shoot length, multiplication coefficient, and hyperhydricity values (Figures 1E, 5). However, the shoots cultured in TIS with *β*-A grew successfully with low sucrose supplementation, suggesting that they had developed a photoautotrophic behavior (Figures 1F, 5). For SL and H, an interaction was detected for the culture media and the percentage of sucrose (*p* = 0.042 and *p* = 0.020), but not for NS and MC (*p* = 0.243 and *p* = 0.081). To further explore the effect of sucrose supplementation on cannabis proliferation, we cultured Beatriz and Mati explants in RITA® and in jars with *β*-A medium supplemented with 0.5 and 0% sucrose. The absence of sucrose prevented any proliferation and finally caused the death of all explants. An addition of 0.5% sucrose was beneficial for the explants cultured by TIS but not for those cultured in jars, as this concentration did not support the growth of the cultures (Figures 1F–H).

### Effect of the Type of Explant

**Figure 6:**
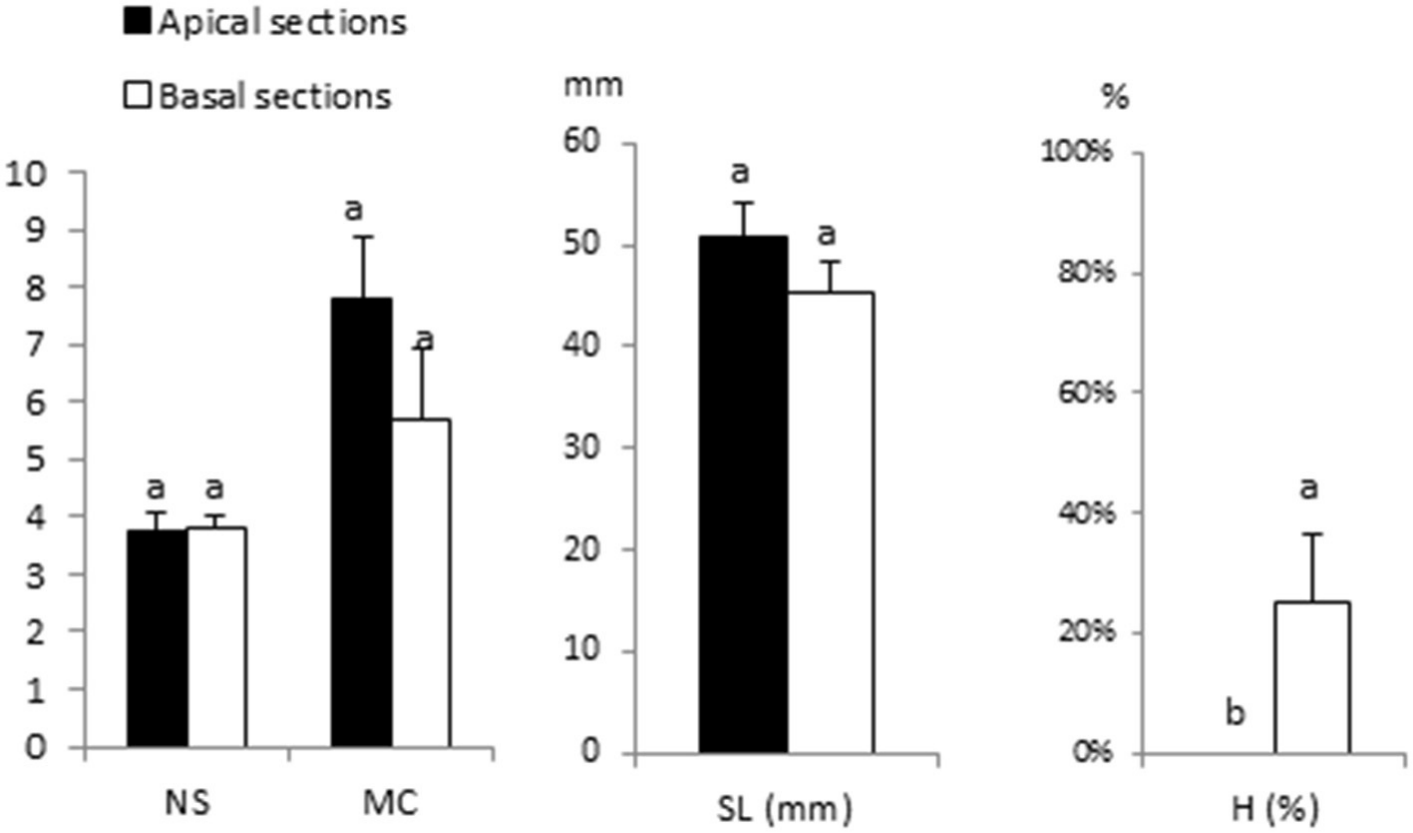
Proliferation of apical vs. basal sections of Beatriz genotype cultured in RITA® vessels in *β*-A medium supplemented with 2-μM metatopoline. Explants (8 per vessel) were immersed for 1 min every 8 h. The data were recorded after 4 weeks of culture. The values represent the mean ± standard error. For each variable, different letters indicate significant differences at *p* < 0.05. NS, number of shoots per explant; MC, multiplication coefficient; SL, length of the longest shoot (mm); H, percentage of hyperhydric shoots.

Figure 6 shows the results of culturing 15-mm apical and basal sections of Beatriz in liquid medium in RITA® bioreactors with *β*-A supplemented with 2% sucrose. The apical sections yielded slightly higher NS, MC, and SL values than the basal segments, but these differences were not significant. The basal sections showed a significantly higher percentage of hyperhydricity (Figure 6).

### Effect of Bioreactor Type

**Figure 7:**
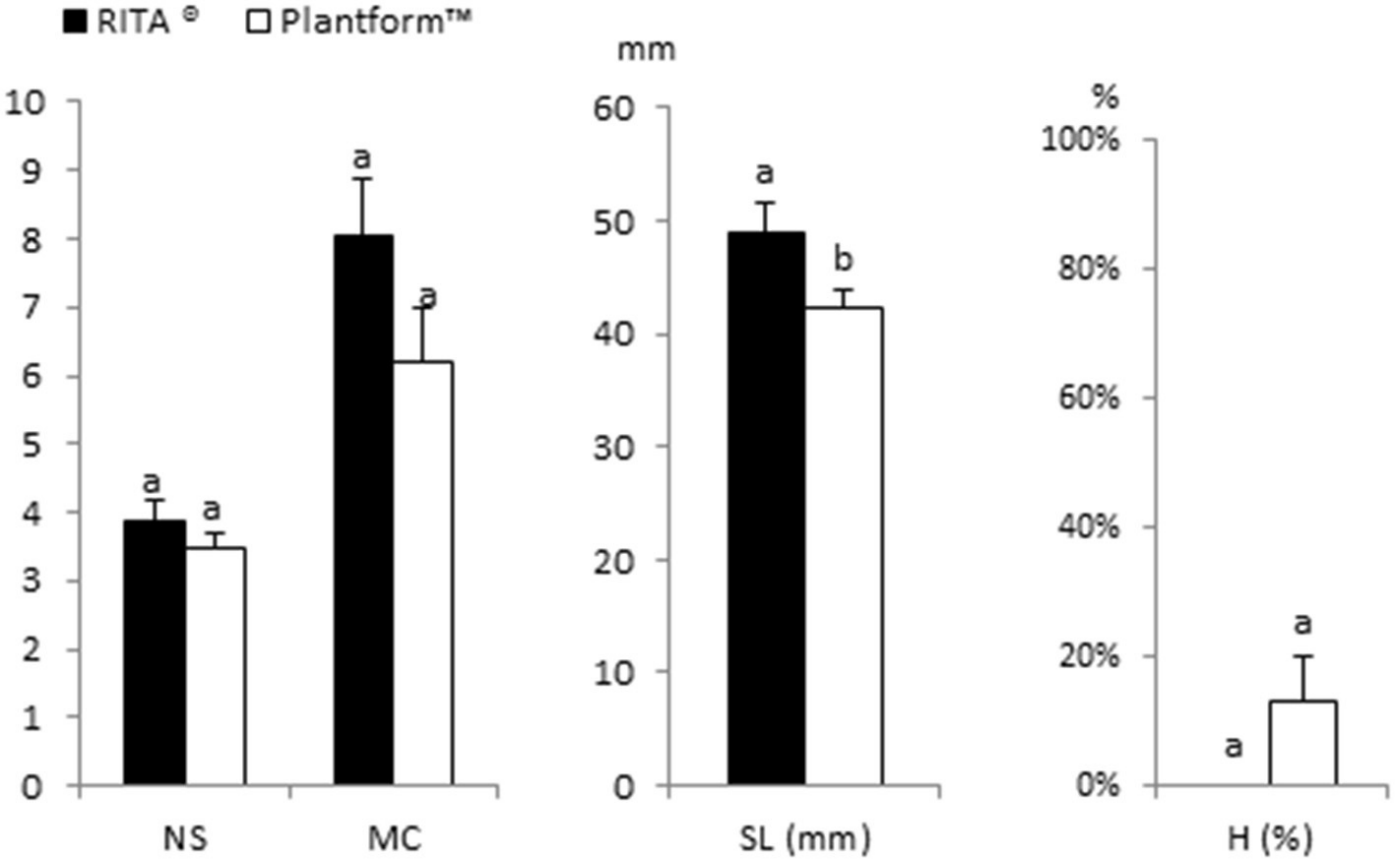
Effect of the type of bioreactor (RITA® and Plantform^TM^) on the proliferation of apical explants of cannabis genotype Beatriz cultured in *β*-A medium with 2 μM metatopoline and 0.5% sucrose. Explants were immersed for 1 min every 8 h. No additional aeration was provided to explants cultured in Plantform™ containers. The data were recorded after 4 weeks of culture. The values represent the mean ± standard error. For each variable, different letters indicate significant differences at *p* < 0.05. NS, number of shoots per explant; MC, multiplication coefficient; SL, length of the longest shoot (mm); H, percentage of hyperhydric shoots.

Figure 7 shows the response of apical explants of Beatriz genotype cultured in RITA® (8 explants) and Plantform™ (24 explants) with *β*-A medium supplemented with 2-μM MT and 0.5% sucrose. In both cases, the explants were immersed in liquid medium for 1 min every 8 h, without an additional aeration in the case of the Plantform™ containers. As shown in Figures 1I, 7, similar NS and MC were obtained for RITA® and Plantform™ bioreactors. The shoots obtained in Plantform™ were significantly longer than the shoots obtained in RITA®, although a slight increase in hyperhydricity was observed in the former bioreactor. Similar results were obtained with Moniek genotype (data not shown).

## Discussion

Studies have been conducted on conventional cannabis micropropagation to produce pathogen-free plants (reviewed in Adhikary et al., 2021; Hesami et al., 2021a; Monthony et al., 2021b). However, a long culture periods, the high production costs per plant, and the poor acclimating efficiency are major obstacles to scaling up for the industrial scale production. Bioreactors helped to overcome these issues with conventional micropropagation in eucalyptus, chestnut, yerba mate, poplar, and hazelnut through the production of vigorous shoots that rooted and acclimated more easily than when cultured with agar (McAlister et al., 2005; Vidal et al., 2015; Arencibia et al., 2017; Luna et al., 2017; Nicholson et al., 2020). In this study, we demonstrated that the use of TIS in the proliferation phase of cannabis micropropagation produced high multiplication coefficients and high-quality shoots.

Beatriz, Moniek, and Mati, the cannabis genotypes in this study were successfully cultured by TIS. The main advantages of the use of liquid instead of semi-solid medium were the shortening of the subculture cycle from 6 to 4 weeks and the use of 4 times less sucrose than in jars, a promising step toward photoautotrophy. Adhikary et al. (2021) suggested that the photoautotrophic bioreactor systems will make the *in vitro* cannabis tissue culture industry more efficient and commercially applicable.

The main factors that influenced cannabis proliferation by TIS were the immersion frequency, the density of explants, the mineral nutrient medium, and the length of the subculture period. For the Beatriz genotype, we further investigated the effect of explant type and bioreactor type. Basal and apical explants in RITA® could be used almost equivalently, although in the conditions applied in this study basal explants produced higher percentages of hyperhydric shoots, as also occurred with apical explants cultured in Plantform™.

Immersion frequency is considered as one of the most important parameters for the efficiency of TIS, and needs to be optimized for each species and each genotype. Low immersion frequencies can decrease the multiplication rates due to a reduction in the supply of nutrients (González et al., 2011) whereas a more frequent contact with the medium can produce more hyperhydric shoots, as reported in pistachio and myrobolan plum (Akdemir et al., 2014; Nasri et al., 2019). In our study, three immersions per day produced good proliferation levels for the three cannabis genotypes, whereas the higher immersion frequencies increased hyperhydricity in all cases. We observed similar trends but detected differences in the absolute proliferation rates among our three genotypes. The genotypic differences are common in conventional micropropagation of many plants in semi-solid medium (Pijut et al., 2011) and in bioreactors, as reported for eucalyptus (McAlister et al., 2005), blueberry (Debnath, 2009), pistachio (Akdemir et al., 2014), and chestnut (Vidal et al., 2015). For genotypes Beatriz and Moniek increasing the frequency of immersion did not significantly affect the NS, and SL, but due to the hyperhydricity caused by this immersion frequency MC decreased (Figures 2A,B). For Mati genotype, with higher explant density, NS and SL increased significantly with six immersions/day, but due to the hyperhydricity caused by this immersion frequency MC did not show any improvement (Figure 2C). Three immersions per day were applied to this genotype in subsequent experiments with lower explant density (Figure 3B) and the proliferation parameters reached values consistent with those showed by Beatriz and Moniek in Figures 2A,B with the same immersion regime. Three immersions per day were therefore used for the rest of the experiments. To apply the lowest immersion frequency has potential additional advantages, such as reducing fungal contamination risk and filter clogging. Similarly, in sugarcane, Mordocco et al. (2009) used the lowest frequency of immersion that did not lead to significant differences to decrease the risk of contamination.

The inoculation density influences the availability of nutrients, gases, and light to individual explants (Georgiev et al., 2014). The optimum density depends on the species (Son et al., 1999), as it is affected by the size of the initial explants, the rate of nutrients consumption and the shape and size of shoots and leaves during their development in the containers. The use of the highest density of explants that do not cause a diminution on growth increases the efficiency of nutrient uptake and laboratory space. The high inoculation densities can reduce the proliferation of the shoots and increase the contamination (Paek et al., 2001), but in some cases as *Curcuma longa* and banana the number of plants per bioreactor could be increased in a 50% percentage maintaining optimal growth responses (Marchant et al., 2021; Uma et al., 2021). In *Cymbopogon citratus* and hazelnut, the low explant density decreased the growth (Quiala et al., 2014; Nicholson et al., 2020). The positive effect of using high explant density in bioreactors could be explained by the growth enhancing activity of substances produced by the explants, as reported for nurse tissues in embryogenic cultures (Hargreaves et al., 2017). In axillary shoots, a certain concentration of the ethylene produced by the explants promotes the development of new buds, both in semi-solid medium and in bioreactors (Nour and Thorpe, 1994; Lai et al., 2005; Mingozzi et al., 2009), although excessive ethylene can cause hyperhydricity (Lai et al., 2005). If too much ethylene is removed with aerations, the use of a higher number of explants could help restore ethylene concentration to a level for bud formation. In our study, we evaluated the different explant densities with the aim of testing this hypothesis in cannabis but preliminary investigations did not support the benefit of increasing explant number in experiments with Mati genotype. Eight or ten explants per RITA® produced numerous and vigorous shoots with large leaves in all the genotypes, indicating an efficient use of medium and headspace.

Murashige and Skoog-based media are the most frequently employed for cannabis multiplication, with reports of its use in semi-solid medium (Richez-Dumanois et al., 1986; Lata et al., 2009a,b, 2016; Grulichova et al., 2017; Mubi et al., 2020; Wróbel et al., 2020), and in thin layer liquid medium (Piunno et al., 2019), although the other alternatives have been proposed, such as IMB4 (Smýkalová et al., 2019) and DKW (Page et al., 2020; Monthony et al., 2021a). Some of the approaches for optimizing the mineral and organic components of the culture medium for a particular plant are the formulations based on the elemental analysis of plant tissues (Nas and Read, 2004) and the use of mathematical methodologies as artificial neural networks. This last strategy has been used for a variety of species (Gago et al., 2011; Akin et al., 2017) including cannabis (Pepe et al., 2021; Hesami et al., 2021b). The absorption of salts and organic compounds such as vitamins, sugars, and plant regulators differ between conventional agar-based medium and liquid medium (Feito et al., 2001; Moncaleán et al., 2003), and for developing the protocols with new culture systems may be necessary to adjust some parameters related with the medium composition. For optimizing cannabis proliferation in TIS, we tested three media based on MS and *β* formulations, which had been used with good results in the previous research with this plant. The Formula *β* used in our study was beneficial for culturing in semi-solid medium several cannabis cultivars (Casano and Grassi, 2009; Codesido et al., 2020), other herbaceous plants such as Chinese foxglove (Yu et al., 2010), potato and various species of orchids (Codesido et al., 2020), and woody plants such as birch, cherry, eucalyptus, wild pear, and willow (Vidal et al., 2018). In our study with TIS, Formula *β* without added vitamins instead of MS–½N with vitamins led to a significant increase in the growth of our three cannabis cultivars. It is worth noting that the shoots propagated with *β*-A by TIS could grow with 0.5% sucrose without showing a decrease in the shoot length or multiplication coefficient regarding the 2% sucrose treatment, suggesting that they had some photoautotrophic behavior. By contrast, the shoots cultured in jars could not grow with this sucrose concentration. These results agree with the reports of Hesami et al. (2021b) and Pepe et al. (2021) on the role of sucrose on different phases of cannabis micropropagation and its interaction with light conditions. These authors recommended the use of 2–3% sucrose for maximizing shoot growth and development in semi-solid medium. Similarly, chestnut and willow shoots did not show successful development in absence of sugar or with low sugar concentrations when cultured with semi-solid medium in closed containers, even if they were placed under high light intensity (Sáez et al., 2016; Gago et al., 2021). Bioreactors with forced ventilation can enhance photosynthesis and eliminate the dependence of exogenous sugars from the media; therefore, decreasing microbial contamination and producing shoots physiologically healthier and better adapted to acclimation than those cultured under photomixotrophic conditions (Xiao et al., 2011; Nguyen et al., 2020). In our study, cannabis shoots cultured in bioreactors grew with 0.5% sucrose but did not grow when the medium was completely devoid of this sugar. *Salix viminalis* shoots were successfully micropropagated in a medium without sugar only when RITA® vessels were placed under high light intensity (150 μmol m^−2^s^−1^) and received carbon dioxide (CO_2_)-enriched air with every immersion (Gago et al., 2021). The use of CO_2_-enriched air to develop autotrophy has been recommended for several authors (Zobayed, 2005; Xiao et al., 2011; Nguyen et al., 2020). These conditions were applied to plum and chestnut to reduce the sucrose concentration when these species were cultured by TIS in RITA® and Plantform™ bioreactors (Gago et al., 2022a,b), and could also represent a useful approach to proliferate cannabis shoots photoautotrophically by TIS.

Two reports on the use of *in vitro* photoautotrophic methods for cannabis propagation have been published (Kodym and Leeb, 2019; Zarei et al., 2021). These studies focused on rooting, rather than on shoot proliferation and did not provide the data on multiplication coefficients. In both cases, the liquid medium was used in a continuous immersion system in which the shoots were inserted in rockwool blocks wetted with nutrient solutions without sugar. Kodym and Leeb (2019) placed the explants under conventional light intensity (70 μmol m^−2^s^−1^) and obtained gas exchanges through forced ventilation with ambient air, whereas Zarei et al. (2021) used a higher light intensity (150 μmol m^−2^s^−1^) and natural ventilation in a CO_2_-enriched chamber. The initial explants used in these two reports were 30–70 mm in size, whereas our shoots were only 15-mm long. As highlighted by Miyashita et al. (1996), under photoautotrophic conditions, an explant must produce a shoot/plantlet using either carbohydrates produced by photosynthesis of the explant or by stored carbohydrates in the explant itself, or both. These authors reported that increasing the explant size and leaf area was beneficial for potato explants cultured without sucrose and with natural ventilation in a CO_2_-enriched chamber (Miyashita et al., 1996). García-Ramírez et al. (2019) reported the photoautotrophic propagation of bamboo by TIS with forced ventilation without CO_2_-enriched air, but their initial explants were larger than the cannabis sections we used in this study. The use of larger explants with more storage capacity could enable the photoautotrophic growth of cannabis and to be evaluated in future TIS experiments using either ambient or CO_2_-enriched air.

The bioreactors used for TIS differ in the type of material, size and shape, and the designs may influence their suitability for the micropropagation of specific species. In this study, we tested the proliferation of cannabis in Plantform™ vessels applying the protocol optimized in RITA®. Apical shoots were cultured for 4 weeks in *β*-A medium supplemented with 0.5% sucrose using 1-min immersion every 4 h. Plantform™ containers are bigger, and they have greater headspace and more capacity for the cultivation medium (Welander et al., 2014). Although Plantform™ bioreactors allow additional ventilation independently of immersion, in this study, we maintained the aeration regime of RITA® vessels. Similar growth and multiplication coefficients were observed in both bioreactors, although a slight increase of hyperhydricity was detected in Plantform™. In our previous studies on chestnut (Vidal et al., 2015), willow (Regueira et al., 2018), and alder (San José et al., 2020), we observed minimal hyperhydricity in those bioreactors, and the shoots showed significantly increased shoot length and multiplication coefficient than those cultured in RITA® vessels. In those experiments, Plantform™ vessels received additional aerations of 1 min every hour, whereas in this study, no additional aeration was applied to cannabis explants. Increasing the air supply inside the bioreactors can reduce the internal humidity and favor gas exchange between the plant and the surrounding environment, preventing hyperhydricity and increasing normal metabolism of plant tissues (Chakrabarty et al., 2003; Kozai and Kubota, 2005; Roels et al., 2006). Forced ventilation improved the propagation of many species (Xiao et al., 2011) thus applying additional aeration cycles to the Plantform™ containers could be beneficial for cannabis proliferation in TIS. This strategy would take more advantage of the specific features of both types of containers. The larger size of Plantform™ bioreactors enables up to a 3-fold increase in the number of explants that could be processed at a time, making them more suitable for a large-scale propagation. In contrast, RITA® bioreactors contain fewer explants but require less medium per explant than Plantform™ vessels. These containers may be the most useful ones when the plant material is scarce or for initiating and maintaining cannabis shoots in liquid media.

## Conclusions

This is the first report on the use of TIS systems in *C. sativa* micropropagation, with the results of this study providing new perspectives and opportunities for the mass propagation of this challenging species. This method provides a simple and efficient *in vitro* propagation system for the large-scale multiplication of cannabis, which shortens the culture period and reduces the use of sucrose. Beatriz, Moniek, and Mati genotypes were successfully cultured in liquid medium using commercial RITA® temporary immersion bioreactors and Plantform™, which showed promising results in the two genotypes in which was tested (Beatriz and Moniek). We propose a protocol in which apical cannabis explants (size, 15 mm) can be cultured for 4 weeks in RITA® and Plantform™ bioreactors. Eight explants per RITA® and 24 per Plantform™, together with 1-min immersion every 8 h in *β*-A medium without vitamins and with 0.5% sucrose produced high multiplication coefficients and vigorous shoots for the three genotypes. Future research could focus on the following: (i) optimization of additional aerations in Plantform™ bioreactors, (ii) investigation of the roles of light intensity and the supplementation of CO_2_-enriched air on the photoautotrophic behavior of cannabis, and (iii) the effect of sucrose supplementation, light and gas composition on the rooting, and acclimation response of cannabis shoots produced in bioreactors.

## Data Availability Statement

The datasets presented in this article are not readily available because some restrictions regarding the confidential agreement between CSIC and private company. Requests to access the datasets should be directed to nieves@iiag.csic.es.

## Author Contributions

NV, CS, and VC conceived the study. SR and NV conducted the main experiments. CF-V, JG, VC, CS, and NV collaborated in funding acquisition and provided resources. SR wrote the original draft. SR, NV, and JG collaborated in review and editing of manuscripts. NV and CS supervised the whole process. All authors contributed to the article and approved the final version.

## Funding

This work was supported by a research contract from Phytoplant Research (Ref. 20190548) and by the Xunta de Galicia (Spain) through the projects IN607A 2021/06 and Contrato Programa 2021 (AGI/CSIC I+D+I 2021, Ref- ACAM 20210200033).

## Conflict of Interest

JG, CF-V, and VC are employed by Phytoplant Research SLU. The remaining authors declare that the research was conducted in the absence of any commercial or financial relationships that could be construed as a potential conflict of interest.

## Publisher's Note

All claims expressed in this article are solely those of the authors and do not necessarily represent those of their affiliated organizations, or those of the publisher, the editors and the reviewers. Any product that may be evaluated in this article, or claim that may be made by its manufacturer, is not guaranteed or endorsed by the publisher.

## References

[B1] Adhikary D. Kulkarni M. El-Mezawy A. Mobini S. Elhiti M. Gjuric R.. (2021). Medical Cannabis and industrial hemp tissue culture: present status and future potential. Front. Plant Sci. 12:627240. 10.3389/fpls.2021.627240 33747008 PMC7968383

[B2] Aitken-Christie J. (1991). Automation, in Micropropagation, eds Debergh P. C. Zimmerman R. H. (Dordrecht: Springer), 363–388. 10.1007/978-94-009-2075-0_23

[B3] Akdemir H. Süzerer V. Onay A. Tilkat E. Ersali Y. Çiftçi Y. O. (2014). Micropropagation of the pistachio and its rootstocks by temporary immersion system. Plant Cell. Tissue Organ Cult. 117, 65–76. 10.1007/s11240-013-0421-0

[B4] Akin M. Eyduran E. Reed B. M. (2017). Use of RSM and CHAID data mining algorithm for predicting mineral nutrition of hazelnut. Plant Cell Tissue Organ Cult. 128, 303–316. 10.1007/s11240-016-1110-6

[B5] Albarrán J. Bertrand B. Lartaud M. Etienne H. (2005). Cycle characteristics in a temporary immersion bioreactor affect regeneration, morphology, water and mineral status of coffee (*Coffea arabica*) somatic embryos. Plant Cell. Tissue Organ Cult. 81, 27–36. 10.1007/s11240-004-2618-8

[B6] Aliferis K. A. Bernard-Perron D. (2020). Cannabinomics: application of metabolomics in Cannabis (*Cannabis sativa* L.) research and development. Front. Plant Sci. 11:554. 10.3389/fpls.2020.00554 32457786 PMC7225349

[B7] Arencibia A. D. Gómez A. Poblete M. Vergara C. (2017). High performance micropropagation of dendroenergetic poplar hybrids in photomixotrophic Temporary Immersion Bioreactors (TIBs). Ind. Crop. Prod. 96, 102–109. 10.1016/j.indcrop.2016.11.065

[B8] Campbell L. G. Naraine S. G. U. Dusfresne J. (2019). Phenotypic plasticity influences the success of clonal propagation in industrial pharmaceutical *Cannabis sativa*. PLoS ONE 14:e0213434. 10.1371/journal.pone.0213434 30883573 PMC6422331

[B9] Casano S. Grassi G. (2009). “Evaluation of media for hemp (Cannabis sativa L.) in vitro propagation”, in Comunicazioni e riassunti del Convegno Nazionale La micropropagazione in Italia: stato attuale e prospettive future. Un incontro tra operatori del settore e della ricerca, Legnaro, Italia, 20-21 November 2008, Italus Hortus 16, 109–112.27108324

[B10] Chakrabarty D. Hahn E. J. Yoon Y. J. Paek K. Y. (2003). Micropropagation of apple rootstock M.9 EMLA using bioreactor. J. Hortic. Sci. Biotechnol. 78, 605–609. 10.1080/14620316.2003.11511671

[B11] Chandra S. Lata H. Khan I. A. ElSohly M. A. (2017). “*Cannabis sativa* L.: Botany and horticulture,” in *Cannabis sativa L.* - Botany and Biotechnology (Cham: Springer), 79–100. 10.1007/978-3-319-54564-6_3

[B12] Codesido V. Meyer S. Casano S. (2020). Influence of media composition and genotype for successful *Cannabis sativa* L. *in vitro* introduction. Acta Hortic. 1285, 75–80. 10.17660/ActaHortic.2020.1285.12

[B13] Cuenca B. Sánchez C. Aldrey A. Bogo B. Blanco B. Correa B.. (2017). Micropropagation of axillary shoots of hybrid chestnut (*Castanea sativa* × *C. crenata)* in liquid medium in a continuous immersion system. Plant Cell. Tissue Organ Cult. 131, 307–320. 10.1007/s11240-017-1285-5

[B14] Debnath S. C. (2009). A scale-up system for Lowbush Blueberry micropropagation using a bioreactor. HortScience 44, 1962–1966. 10.21273/HORTSCI.44.7.1962

[B15] Etienne H. Berthouly M. (2002). Temporary immersion systems in plant micropropagation. Plant Cell. Tissue Organ Cult. 69, 215–231. 10.1023/A:1015668610465

[B16] Feito I. González A. Centeno M. L. Fernández B. Rodríguez A. (2001). Transport and distribution of benzyladenine in *Actinidia deliciosa* explants cultured in liquid and solid media. Plant Physiol. Biochem. 39, 909–916. 10.1016/S0981-9428(01)01309-2

[B17] Gago D. Bernal M. Á. Sánchez C. Aldrey A. Cuenca B. Christie C. B.. (2022a). Effect of sucrose on growth and stress status of *Castanea sativa* x *C. crenata* shoots cultured in liquid medium. Plants 11:965. 10.3390/plants11070965 35406943 PMC9003454

[B18] Gago D. Sánchez C. Aldrey A. Christie C. B. Bernal M. Á. Vidal N. (2022b). Micropropagation of Plum (*Prunus domestica* L.) in bioreactors using photomixotrophic and photoautotrophic conditions. Horticulturae 8:286. 10.3390/horticulturae8040286

[B19] Gago D. Vilavert S. Bernal M. Á. Sánchez C. Aldrey A. Vidal N. (2021). The effect of sucrose supplementation on the micropropagation of *Salix viminalis* L. shoots in semisolid medium and temporary immersion bioreactors. Forests 12:1408. 10.3390/f12101408

[B20] Gago J. Pérez-Tornero O. Landín M. Burgos L. Gallego P. P. (2011). Improving knowledge of plant tissue culture and media formulation by neurofuzzy logic: a practical case of data mining using apricot databases. J. Plant Physiol. 168, 1858–1865. 10.1016/j.jplph.2011.04.008 21676490

[B21] García-Ramírez Y. Barrera G. P. Freire-Seijo M. Barbón R. Concepción-Hernández M. Mendoza-Rodríguez M. F.. (2019). Effect of sucrose on physiological and biochemical changes of proliferated shoots of *Bambusa vulgaris* Schrad. Ex Wendl in temporary immersion. Plant Cell. Tissue Organ Cult. 137, 239–247. 10.1007/s11240-019-01564-z

[B22] Georgiev V. Schumann A. Pavlov A. Bley T. (2014). Temporary immersion systems in plant biotechnology. Eng. Life Sci. 14, 607–621. 10.1002/elsc.201300166

[B23] González R. Ríos D. Avilés F. Sánchez-Olate M. (2011). Multiplicación in vitro de *Eucalyptus globulus* mediante sistema de inmersión temporal. Bosque 32, 147–154. 10.4067/S0717-92002011000200005

[B24] Grulichova M. Mendel P. Lalge A. B. Slamova N. Trojan V. Vyhnanek T.. (2017). “Effect of different phytohormones on growth and development of micropropagated *Cannabis sativa* L.,” in Conference: MendelNet 2017 - Proceedings of 24th International PhD Students Conference (ISBN 978-80-7509-529-9) At: Mendel University in Brno, Czech RepublicVolume 24, 618–623.

[B25] Hargreaves C. Reeves C. Gough K. Montalbán I. A. Low C. van Ballekom S.. (2017). Nurse tissue for embryo rescue: testing new conifer somatic embryogenesis methods in a F1 hybrid pine. Trees 31, 273–283. 10.1007/s00468-016-1482-6

[B26] Hesami M. Baiton A. Alizadeh M. Pepe M. Torkamaneh D. Jones A. M. P. (2021a). Advances and perspectives in tissue culture and genetic engineering of cannabis. Int. J. Mol. Sci. 22:5671. 10.3390/ijms22115671 34073522 PMC8197860

[B27] Hesami M. Pepe M. Alizadeh M. Rakei A. Baiton A. Jones A. M. P. (2020). Recent advances in cannabis biotechnology. Ind. Crops Prod. 158:113026. 10.1016/j.indcrop.2020.113026

[B28] Hesami M. Pepe M. Monthony A. S. Baiton A. Jones A. M. P. (2021b). Modeling and optimizing *in vitro* seed germination of industrial hemp (*Cannabis sativa* L.). *Ind. Crops Prod*. 170:113753. 10.1016/j.indcrop.2021.113753

[B29] Kodym A. Leeb C. J. (2019). Back to the roots: protocol for the photoautotrophic micropropagation of medicinal Cannabis. Plant Cell. Tissue Organ Cult. 138, 399–402. 10.1007/s11240-019-01635-1 31404230 PMC6660493

[B30] Kovalchuk I. Pellino M. Rigault P. Van Velzen R. Ebersbach J. Ashnest J. R.. (2020). The genomics of Cannabis and its close relatives. Annu. Rev. Plant Biol. 71, 713–739. 10.1146/annurev-arplant-081519-040203 32155342

[B31] Kozai T. Kubota C. (2005). “In Vitro root zone environments and their effects on growth and development of plants”, in Photoautotrophic (sugar-free medium) Micropropagation as a New Micropropagation and Transplant Production System, eds Kozai T. Afreen F. Zobayed S. (Dordrecht: Springer), 53–60. 10.1007/1-4020-3126-2_5

[B32] Lai C. C. Lin H. M. Nalawade S. M. Fang W. Tsay H. S. (2005). Hyperhydricity in shoot cultures of *Scrophularia yoshimurae* can be effectively reduced by ventilation of culture vessels. J. Plant Physiol. 162, 355–361. 10.1016/j.jplph.2004.07.015 15832688

[B33] Lata H. Chandra S. Khan I. ElSohly M. (2010). *Cannabis sativa* L. Micropropagation in temporary immersion bioreactor system. Planta Med. 76:P9. 10.1055/s-0030-1251774

[B34] Lata H. Chandra S. Khan I. ElSohly M. A. (2009b). Thidiazuron-induced high-frequency direct shoot organogenesis of *Cannabis sativa* L. In Vitro Cell. Dev. Biol. Plant 45, 12–19. 10.1007/s11627-008-9167-5

[B35] Lata H. Chandra S. Khan I. A. Elsohly M. A. (2009a). Propagation through alginate encapsulation of axillary buds of *Cannabis sativa* L. *-* An important medicinal plant. Physiol. Mol. Biol. Plants 15, 79–86. 10.1007/s12298-009-0008-8 23572915 PMC3550375

[B36] Lata H. Chandra S. Techen N. Khan I. A. ElSohly M. A. (2016). *In vitro* mass propagation of *Cannabis sativa* L.: a protocol refinement using novel aromatic cytokinin meta-topolin and the assessment of eco-physiological, biochemical and genetic fidelity of micropropagated plants. J. Appl. Res. Med. Aromat. Plants 3, 18–26. 10.1016/j.jarmap.2015.12.001

[B37] Leyva-Ovalle O. R. Bello-Bello J. J. Murguía-González J. Núñez-Pastrana R. Ramírez-Mosqueda M. A. (2020). Micropropagation of *Guarianthe skinneri* (Bateman) Dressler et W. E. Higging in Temporary Immersion Systems. 3 Biotech 10:26. 10.1007/s13205-019-2010-3 31938685 PMC6942062

[B38] Luna C. V. Gonzalez A. M. Mroginski L. A. Sansberro P. A. (2017). Anatomical and histological features of *Ilex paraguariensis* leaves under different *in vitro* shoot culture systems. Plant Cell Tiss. Organ. Cult. 129, 457–467. 10.1007/s11240-017-1191-x

[B39] Mallón R. Covelo P. Vieitez A. M. (2012). Improving secondary embryogenesis in *Quercus robur*: application of temporary immersion for mass propagation. Trees Struct. Funct. 26, 731–741. 10.1007/s00468-011-0639-6

[B40] Mallón R. Vieitez A. M. Vidal N. (2013). High-efficiency Agrobacterium-mediated transformation in *Quercus robur*: selection by use of a temporary immersion system and assessment by quantitative PCR. Plant Cell Tissue Organ Cult. 114, 171–185. 10.1007/s11240-013-0313-3

[B41] Mancilla-Álvarez E. Pérez-Sato J. A. Núñez-Pastrana R. Spinoso-Castillo J. L. Bello-Bello J. J. (2021). Comparison of different semi-automated bioreactors for *in vitro* propagation of taro (*Colocasia esculenta* L. *schott*). Plants 10:1010. 10.3390/plants10051010 34069416 PMC8159139

[B42] Marchant M. J. Molina P. Montecinos M. Guzmán L. Balada C. Fassio C.. (2021). *In vitro* propagation of Easter Island *Curcuma longa* from rhizome explants using temporary immersion system. Agronomy 11:2121. 10.3390/agronomy11112121

[B43] McAlister B. Finnie J. Watt M. P. Blakeway F. (2005). Use of the temporary immersion bioreactor system (RITA®) for production of commercial *Eucalyptus* clones in Mondi Forests (SA). Plant Cell Tissue Organ Cult. 81, 347–358. 10.1007/s11240-004-6658-x

[B44] Melviana A. C. Esyanti R. R. Mel M. Setyobudi R. H. (2021). “Biomass enhancement of *Stevia rebaudiana* bertoni shoot culture in temporary immersion system (TIS) RITA® bioreactor optimized in two different immersion periods,” in E3S Web of Conferences.

[B45] Mingozzi M. Montello P. Merkle S. (2009). Adventitious shoot regeneration from leaf explants of eastern cottonwood (*Populus deltoides*) cultured under photoautotrophic conditions. Tree Physiol. 29, 333–343. 10.1093/treephys/tpn029 19203957

[B46] Miyashita Y. Kitaya Y. Kubota C. Kozai T. (1996). Photoautotrophic growth of potato plantlets as affected by explant leaf area, fresh weight and stem length. Sci. Hortic. 65, 199–202. 10.1016/0304-4238(96)00877-1

[B47] Moncaleán P. Cañal M. J. Fernández H. Fernández B. Rodríguez A. (2003). Nutritional and gibberellic acid requirements in kiwifruit vitroponic cultures. In Vitro Cell. Dev. Biol. Plant 39, 49–55. 10.1079/IVP2002371

[B48] Monja-Mio K. M. Olvera-Casanova D. Herrera-Alamillo M. Sánchez-Teyer F. L. Robert M. L. (2021). Comparison of conventional and temporary immersion systems on micropropagation (multiplication phase) of *Agave angustifolia Haw*. ‘Bacanora.' 3 Biotech 11:77. 10.1007/s13205-020-02604-8 33505832 PMC7810801

[B49] Monthony A. S. Bagheri S. Zheng Y. Jones A. M. P. (2021a). Flower power: floral reversion as a viable alternative to nodal micropropagation in *Cannabis sativa*. In Vitro Cell. Dev. Biol. Plant. 57, 1018–1030. 10.1007/s11627-021-10181-5

[B50] Monthony A. S. Page S. R. Hesami M. Jones A. M. P. (2021b). The past, present and future of *Cannabis sativa* tissue culture. Plants 10:185. 10.3390/plants10010185 33478171 PMC7835777

[B51] Mordocco A. M. Brumbley J. A. Lakshmanan P. (2009). Development of a temporary immersion system (RITA®) for mass production of sugarcane (*Saccharum* spp. interspecific hybrids). In Vitro Cell. Dev. Biol. Plant 45, 450–457. 10.1007/s11627-008-9173-7

[B52] Mubi S. M. Svetik S. FlaJ¡Man M. Murovec J. (2020). *In vitro* tissue culture and genetic analysis of two high-cbd medical cannabis (*Cannabis sativa* L.) breeding lines. Genetika 52, 925–941. 10.2298/GENSR2003925M

[B53] Murashige T. Skoog F. (1962). A revised medium for rapid growth and bio assays with tobacco tissue cultures. Physiol. Plant. 15, 473–497. 10.1111/j.1399-3054.1962.tb08052.x

[B54] Nas M. N. Read P. E. (2004). A hypothesis for the development of a defined tissue culture medium of higher plants and micropropagation of hazelnuts. Sci Hortic. 101, 189–200. 10.1016/j.scienta.2003.10.004

[B55] Nasri A. Baklouti E. Ben Romdhane A. Maalej M. Schumacher H. M. Drira N.. (2019). Large-scale propagation of Myrobolan (*Prunus cerasifera*) in RITA® bioreactors and ISSR-based assessment of genetic conformity. Sci. Hortic. 245, 144–153. 10.1016/j.scienta.2018.10.016

[B56] Nguyen Q. T. Xiao Y. Kozai T. (2020). “Photoautotrophic micropropagation,” in Plant Factory-An Indoor Vertical Farming System for Efficient Quality Food Production, 2nd Edn, eds Kozai T. Niu G. Takagaki M. (New York, NY: Elsevier, Academic Press), 333–346. 10.1016/B978-0-12-816691-8.00023-6

[B57] Nicholson J. Shukla M. R. Saxena P. K. (2020). In vitro rooting of hybrid hazelnuts (Corylus avellana × Corylus americana) in a temporary immersion system. Botany. 98, 343–352. 10.1139/cjb-2019-0206

[B58] Nour K. A. Thorpe T. A. (1994). The effect of the gaseous state on bud induction and shoot multiplication *in vitro* in Eastern white cedar. Physiol. Plant 90, 163–172. 10.1111/j.1399-3054.1994.tb02207.x

[B59] Oultram J. M. J. Pegler J. L. Bowser T. A. Ney L. J. Eamens A. L. Grof C. P. L. (2021). *Cannabis sativa*: interdisciplinary strategies and avenues for medical and commercial progression outside of CBD and THC. Biomedicines 9:234. 10.3390/biomedicines9030234 33652704 PMC7996784

[B60] Paek K. Y. Hahn E. J. Son S. H. (2001). Application of bioreactors for large-scale micropropagation systems of plants. In Vitro Cell. Dev. Biol. Plant 37, 149–157. 10.1007/s11627-001-0027-929981112

[B61] Page S. Monthony A. Jones A. M. P. (2020). DKW basal salts improve micropropagation and callogenesis compared to MS basal salts in multiple commercial cultivars of *Cannabis sativa*. Botany 99, 269–279. 10.1139/cjb-2020-0179

[B62] Pepe M. Hesami M. Small F. Jones A. M. P. (2021). Comparative analysis of machine learning and evolutionary optimization algorithms for precision micropropagation of *Cannabis sativa*: prediction and validation of *in vitro* shoot growth and development based on the optimization of light and carbohydrate sources. Front. Plant Sci. 12:757869. 10.3389/fpls.2021.757869 34745189 PMC8566924

[B63] Pijut P. M. Lawson S. S. Michler C. H. (2011). Biotechnological efforts for preserving and enhancing temperate hardwood tree biodiversity, health, and productivity. In Vitro Cell. Dev. Biol. Plant 47, 123–147. 10.1007/s11627-010-9332-5

[B64] Piunno K. F. Golenia G. Boudko E. A. Downey C. Maxwell A. (2019). Regeneration of shoots from immature and mature inflorescences of *Cannabis sativa*. Can. J. Plant Sci. 99, 556–559. 10.1139/cjps-2018-0308

[B65] Quiala E. Barbón R. Capote A. Pérez-Alonso N. Chávez M. de Feria M.. (2014). Scaling-up the biomass production of *Cymbopogon citratus* L. in temporary immersion system. Biotecnol. Veg. 14, 67–71.

[B66] Regueira M. Rial E. Blanco B. Bogo B. Aldrey A. Correa B.. (2018). Micropropagation of axillary shoots of *Salix viminalis* using a temporary immersion system. Trees Struct. Funct. 32, 61–71. 10.1007/s00468-017-1611-x

[B67] Richez-Dumanois C. Braut-Boucher F. Cosson L. Paris M. (1986). Multiplication végétative *in vitro* du chanvre (*Cannabis sativa* L.). Application à la conserva- tion des clones sélectionnés. Agronomie 6, 487–495. 10.1051/agro:19860510

[B68] Roels S. Noceda C. Escalona M. Canal M. J. Rodriguez R. Debergh P. (2006). The effect of headspace renewal in a Temporary Immersion Bioreactor on plantain (Musa AAB) shoot proliferation and quality. Plant Cell Tissue Organ Cult. 84, 155–163. 10.1007/s11240-005-9013-y

[B69] Sáez P. L. Bravo L. A. Sánchez-Olate M. Bravo P. B. Ríos D. G. (2016). Effect of photon flux density and exogenous sucrose on the photosynthetic performance during *in vitro* culture of *Castanea sativa*. Am. J. Plant Sci. 7, 2087–2105. 10.4236/ajps.2016.714187

[B70] San José M. C. Blázquez N. Cernadas M. J. Janeiro L. V. Cuenca B. Sánchez C.. (2020). Temporary immersion systems to improve alder micropropagation. Plant Cell Tissue Organ Cult. 143, 265–275. 10.1007/s11240-020-01937-9

[B71] Smýkalová I. Vrbová M. Cvečková M. Plačková L. Žukauskaite A. Zatloukal M.. (2019). The effects of novel synthetic cytokinin derivatives and endogenous cytokinins on the *in vitro* growth responses of hemp (*Cannabis sativa* L.) explants. Plant Cell Tissue Organ Cult. 139, 381–394. 10.1007/s11240-019-01693-5

[B72] Son S. H. Park S. M. Park S. Y. Kwon O. W. Paek S.-Y. (1999). Large-scale culture of plant cell and tissue by bioreactor system. J. Plant Biotechnol. 1, 1–7.

[B73] Steingroewer J. Bley T. Georgiev V. Ivanov I. Lenk F. Marchev A.. (2013). Bioprocessing of differentiated plant *in vitro* systems. Eng. Life Sci. 13, 26–38. 10.1002/elsc.201100226

[B74] Uma S. Karthic R. Kalpana S. Backiyarani S. Saraswathi M. S. (2021). A novel temporary immersion bioreactor system for large scale multiplication of banana (Rasthali AAB—Silk). Sci Rep 11:20371. 10.1038/s41598-021-99923-4 34645934 PMC8514489

[B75] Valleriani J. (2020). Identity narratives in the face of market competition: the emerging legal medical cannabis market in Canada. Drugs Educ. Prev. Policy 27, 37–48. 10.1080/09687637.2018.1531828

[B76] Vidal N. Blanco B. Cuenca B. (2015). A temporary immersion system for micropropagation of axillary shoots of hybrid chestnut. Plant Cell Tissue Organ Cult. 123, 229–243. 10.1007/s11240-015-0827-y

[B77] Vidal N. Rico S. Casano S. Codesido V. Sánchez C. (2018). Low cost media for improving *in vitro* propagation of woody plant species, in 5th International Conference of the IUFRO Working Party 2.09.02. Somatic Embryogenesis and Other Vegetative Propagation Technologies. Clonal Trees in The Bioeconomy Age: Opportunities and Challenges; Coimbra, Portugal; 10-15 September 2018. Oral presentation. Book of Abstracts pp 60.

[B78] Vidal N. Sánchez C. (2019). Use of bioreactor systems in the propagation of forest trees. Eng. Life Sci. 19, 896–915. 10.1002/elsc.201900041 32624981 PMC6999064

[B79] Villegas-Sánchez E. Macías-Alonso M. Osegueda-Robles S. Herrera-Isidrón L. Nuñez-Palenius H. González-Marrero J. (2021). *In vitro* culture of *Rosmarinus officinalis* L. in a temporary immersion system: influence of two phytohormones on plant growth and carnosol production. Pharmaceuticals 14:747. 10.3390/ph14080747 34451844 PMC8398425

[B80] Welander M. Persson J. Asp H. Zhu L. H. (2014). Evaluation of a new vessel system based on temporary immersion system for micropropagation. Sci. Hortic. 179, 227–232. 10.1016/j.scienta.2014.09.035

[B81] Weremczuk-Jezyna I. Lisiecki P. Gonciarz W. Kuzma L. Szemraj M. Chmiela M.. (2020). Transformed shoots of *Dracocephalum forrestii* W.W. Smith from different bioreactor systems as a rich source of natural phenolic compounds. Molecules 25:4533. 10.3390/molecules25194533 33022943 PMC7583972

[B82] Wróbel T. Dreger M. Wielgus K. Słomski R. (2020). Modified nodal cuttings and shoot tips protocol for rapid regeneration of *Cannabis sativa* L. J. Nat. Fibers. 19, 536–545. 10.1080/15440478.2020.1748160

[B83] Xiao Y. Niu G. Kozai T. (2011). Development and application of photoautotrophic micropropagation plant system. Plant Cell Tissue Organ Cult. 105, 149–158. 10.1007/s11240-010-9863-9

[B84] Yu M. Mwafulirwa L. D. Cullum J. Bayley J. (2010). Establishment of an efficient protocol for rapid propagation of Chinese foxglove (*Rehmannia angulata*). Med. Plants Int. J. Phytomedicines Relat. Ind. 2, 103–110. 10.5958/j.0975-4261.2.2.016

[B85] Zarei A. Behdarvandi B. Tavakouli Dinani E. Maccarone J. (2021). Cannabis sativa L. photoautotrophic micropropagation: a powerful tool for industrial scale *in vitro* propagation. In Vitro Cell. Dev. Biol. Plant 57, 932–941. 10.1007/s11627-021-10167-3

[B86] Zobayed S. M. A. (2005). Ventilation in micropropagation, in Photoautotrophic (sugar-free medium) Micropropagation as a New Micropropagation and Transplant Production System (Dordrecht: Springer), 147–186. 10.1007/1-4020-3126-2_9

